# Epidemiological Description and Detection of Antimicrobial Resistance in Various Aquatic Sites in Marseille, France

**DOI:** 10.1128/spectrum.01426-22

**Published:** 2023-03-28

**Authors:** Sabah El-Sawalhi, Océane Revol, Amanda Chamieh, Alexandre Lacoste, Alexandre Annessi, Bernard La Scola, Jean-Marc Rolain, Isabelle Pagnier

**Affiliations:** a Aix-Marseille Université, IRD, APHM, MEPHI, Faculté de Médecine et de Pharmacie, Marseille CEDEX 05, France; b IHU Méditerranée Infection, Marseille CEDEX 05, France; c Bataillon des Marins Pompiers de Marseille, CIS BMPM, Marseille, France; McGill University

**Keywords:** aquatic environment, carbapenemase, ESBL, antibiotic resistance

## Abstract

Antibiotic resistance is a worldwide public health concern and has been associated with reports of elevated mortality. According to the One Health concept, antibiotic resistance genes are transferrable to organisms, and organisms are shared among humans, animals, and the environment. Consequently, aquatic environments are a possible reservoir of bacteria harboring antibiotic resistance genes. In our study, we screened water and wastewater samples for antibiotic resistance genes by culturing samples on different types of agar media. Then, we performed real-time PCR to detect the presence of genes conferring resistance to beta lactams and colistin, followed by standard PCR and gene sequencing for verification. We mainly isolated *Enterobacteriaceae* from all samples. In water samples, 36 Gram-negative bacterial strains were isolated and identified. We found three extended-spectrum β-lactamase (ESBL)-producing bacteria—Escherichia coli and Enterobacter cloacae strains—harboring the CTX-M and TEM groups. In wastewater samples, we isolated 114 Gram-negative bacterial strains, mainly E. coli, Klebsiella pneumoniae, Citrobacter freundii and Proteus mirabilis strains. Forty-two bacterial strains were ESBL-producing bacteria, and they harbored at least one gene belonging to the CTX-M, SHV, and TEM groups. We also detected carbapenem-resistant genes, including NDM, KPC, and OXA-48, in four isolates of E. coli. This short epidemiological study allowed us to identify new antibiotic resistance genes present in bacterial strains isolated from water in Marseille. This type of surveillance shows the importance of tracking bacterial resistance in aquatic environments.

**IMPORTANCE** Antibiotic-resistant bacteria are involved in serious infections in humans. The dissemination of these bacteria in water, which is in close contact with human activities, is a serious problem, especially under the concept of One Health. This study was done to survey and localize the circulation of bacterial strains, along with their antibiotic resistance genes, in the aquatic environment in Marseille, France. The importance of this study is to monitor the frequency of these circulating bacteria by creating and surveying water treatments.

## INTRODUCTION

Antimicrobial resistance is a worldwide and multifaceted public health concern, as demonstrated by the spread and emergence of multidrug-resistant (MDR) organisms (1). The increase in MDR infections may be attributed to the increasing prevalence of β-lactamases, including extended-spectrum β-lactamase (ESBL) enzymes, metallo-β-lactamases, and carbapenemases. This subsequently confers resistance to third-generation cephalosporins and then carbapenems (2).

It has been demonstrated that antibiotic resistance may be a process that is transferred between humans, animals, and the environment, especially water (3), giving rise to the concept of One Health. The One Health concept takes on a cooperative and multidisciplinary approach that aims to outline the interrelatedness and optimizes the health outcomes among humans, animals, and their environment (4).

Aquatic environments are a reservoir of antibiotic-resistant bacteria (5) that have increased especially in the presence of antimicrobials (6). Freshwater, mostly rivers and lakes, are well-known environments for the spread and evolution of antibiotic-resistant bacteria (7). Seawater environments also play a major role in the spread and circulation of antibiotics and antibiotic-resistant bacteria (8).

In addition, wastewater also leads to a high and dangerous exposure of bacterial resistance in the environment (9). It has been shown that wastewater provides suitable conditions for the bacterial community to grow, thus leading to the spread of antibiotic resistance (10). The circulation of Escherichia coli isolates producing ESBLs has been reported in several aquatic environments, mostly wastewater (11). In a study conducted on wastewaters in Ivry-sur-Seine in France, several E. coli strains were found to harbor genes, including from the CTX-M and TEM groups (12). In addition, the presence of the CTX-M-1, CTX-M-14, CTX-M-15, and SHV-12 genes was detected in some ESBL-producing bacteria isolated from the wastewater of a hospital located in the city of Besançon, also in France (13). Another study, conducted on hospital wastewater in Clermont-Ferrand, revealed the presence of carbapenemase genes in several genera, notably *Stenotrophomonas*, *Aeromonas*, Acinetobacter, and Pseudomonas spp. (14). The detected antibiotic resistance genes (ARGs) were NDM, VIM, GES, and OXA-48. In addition, colistin resistance genes, such as the *mcr-1*, *mcr-3*, *mcr-4*, and *mcr-5* genes, have been detected in bacterial strains isolated from water (5).

Due to the abundance of bacteria in aquatic environments, it is important to determine whether they are multidrug resistant. Through this demonstration of an integrated One Health approach, we aim to screen several water and wastewater sample sites in Marseille for the presence of ARGs. Consequently, this environmental survey will allow us to identify new ARGs that may be transmissible among humans, animals, and the environment.

## RESULTS

**FIG 1:**
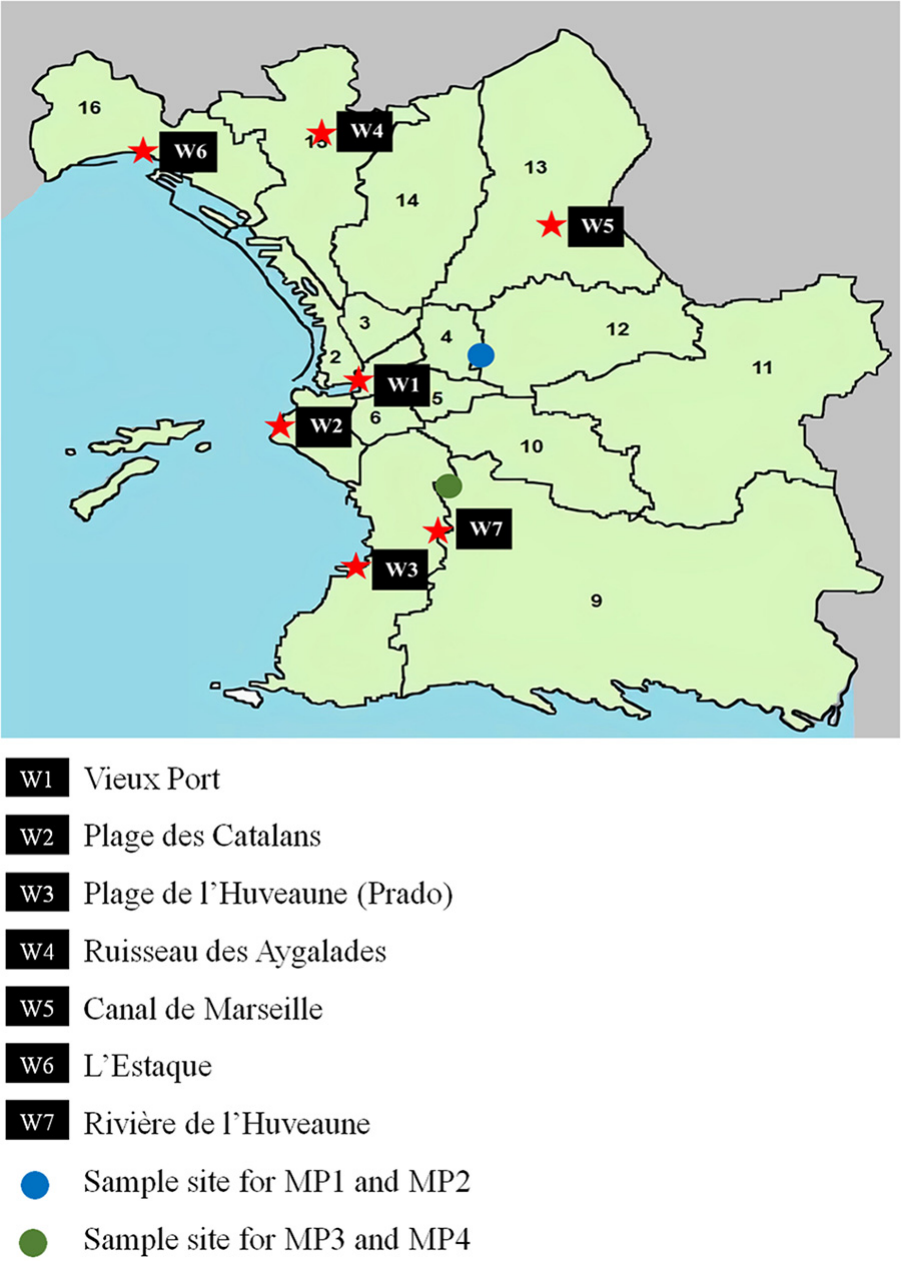
Water sampling sites in Marseille. The red stars represent the sampling site in each area. The blue circle represents the sampling site of the combined sewers. The green circle represents the sampling site of the separated sewers.

Between October and November 2020, we collected 14 samples from seven different aquatic sites (Fig. 1) in Marseille, France. These sites included seawater (W1-Vieux Port, W2-Plage des Catalans, W3-Plage de L’Huveaune, and W6-L’Estaque), freshwater (W4-Ruisseau des Aygalades and W7-L’Huveaune), and drinking water from one of the major sources in Marseille (W5-Canal de Marseille).

We isolated a total of 36 Gram-negative bacteria (Table 1). From the saltwater sites, we identified Serratia marcescens (*n* = 3), Klebsiella oxytoca (*n* = 2), Pseudomonas putida (*n* = 2), Citrobacter braakii (*n* = 1), Acinetobacter ursingii (*n* = 2), and Brevundimonas diminuta (*n* = 1). We did not detect any ARGs in these isolates. From freshwater sites, we identified Enterobacter cloacae (*n* = 5), Escherichia coli (*n* = 2), Pseudomonas putida (*n* = 3), Citrobacter freundii (*n* = 1), and Serratia liquefaciens (*n* = 1). Two isolates obtained from W7 were ESBL producers as determined by antimicrobial susceptibility testing. Real-time and standard PCR testing showed that one E. coli isolate harbored a TEM-98 gene, while another E. cloacae isolate harbored a TEM-214 gene (Table 1). Interestingly, the source of the drinking water from the Canal de Marseille was rich in diversity and the quantity of isolates. We identified Enterobacter cloacae (*n* = 3), Stenotrophomonas maltophilia (*n* = 3), Pseudomonas putida (*n* = 2), Citrobacter gillenii (*n* = 1), Escherichia coli (*n* = 1), and Pseudomonas thivervalensis (*n* = 1). We detected the presence of CTX-M-15 and CTX-M-17 in an E. coli isolate (Table 1).

**TABLE 1:** Identified Gram-negative bacteria isolated from water samples (*n* = 36)

Sampling site (code)	Gram-negative isolates (no.)	Isolate code	ARG(s)^*a*^
Vieux-Port (W1)	Citrobacter braakii (1)	W1a	
	Klebsiella oxytoca (1)	W1b	
	Serratia marcescens (1)	W1c	
	Pseudomonas putida (1)	W1d	
Plage des Catalans (W2)	Klebsiella oxytoca (1)	W2a	
	Serratia marcescens (1)	W2b	
	Pseudomonas putida (1)	W2c	
Plage de l’Huveaune (W3)	Serratia marcescens (1)	W3a	
Ruisseau des Aygalades (W4)	Enterobacter cloacae (2)	W4a	
		W4b	
	Escherichia coli (1)	W4c	
	Pseudomonas putida (2)	W4d	
		W4e	
Canal de Marseille (W5)	Citrobacter gillenii (1)	W5a	
	Enterobacter cloacae (3)	W5b	
		W5c	
		W5d	
	Escherichia coli (1)	W5e	CTX-M-15, CTX-M-17
	Serratia liquefaciens (1)	W5f	
	Pseudomonas putida (2)	W5g	
		W5h	
	Pseudomonas thivervalensis (1)	W5i	
	Stenotrophomonas maltophilia (3)	W5j	
		W5k	
		W5l	
L’Estaque (W6)	Acinetobacter ursingii (2)	W6a	
		W6b	
	Brevundimonas diminuta (1)	W6c	
L’Huveaune (W7)	Citrobacter freundii (1)	W7a	
	Enterobacter cloacae (3)	W7b	
		W7c	
		W7d	TEM-214
	Escherichia coli (1)	W7e	TEM-98
	Serratia liquefaciens (2)	W7f	
		W7g	
	Pseudomonas putida (1)	W7h	

a ARG(s), antibiotic resistance gene(s).

From the wastewater samples, we isolated 114 Gram-negative bacterial strains on different types of media. Table S3 in the supplemental material shows the details of the Gram-negative bacterial strains isolated from the MP1, MP2, MP3, and MP4 membranes of the wastewater samples. A total of 43 strains of the isolated bacteria harbored ARGs (Table 2). Whole-genome sequencing was only performed for the isolated bacterial strains harboring carbapenem-resistant genes. These isolates were E. coli strains: CMP2c, CMP4c, EMP4c, EMP4d, and EMP4e.

**TABLE 2:** ARGs present in the isolates of the wastewater samples (*n* = 43)^*a*^

Membrane	Isolate	Isolate code^*b*^	Antibiotic resistance gene(s)
MP1	E. coli	MMP1a	TEM-206
	E. coli	CMP1a	CTX-M-15, TEM-214
	K. pneumoniae	MMP1c	SHV-187
MP2	C. youngae	MMP2a	TEM-214
	E. coli	MMP2f	TEM-214
	E. coli	CMP2a	CTX-M-27
	E. coli	CMP2b	CTX-M-27, TEM-214
	E. coli	CMP2c*	CTX-M-3, TEM-98, kPC-2
	E. coli	CMP2d	CTX-M-15, TEM-214
	E. coli	CMP2e	CTX-M-15, TEM-214
	E. coli	CMP2f	TEM-206
	P. mirabilis	LMP2b	TEM-217
MP3	E. coli	MMP3c	TEM-206
	E. coli	MMP3d	TEM-214
	E. coli	MMP3f	TEM-206
	K. pneumoniae	MMP3i	SHV-89
	P. mirabilis	MMP3j	TEM-206
	E. coli	MMP3l	TEM-206
	E. coli	CMP3b	CTX-M-15, TEM-206
	E. coli	CMP3c	CTX-M-15, TEM-206
	K. pneumoniae	CMP3d	SHV-12
	K. pneumoniae	CMP3e	SHV-160
MP4	E. coli	MMP4i	TEM-206
	E. coli	MMP4j	TEM-206
	E. coli	MMP4k	TEM-206
	E. coli	MMP4l	SHV-101, TEM-214
	K. pneumoniae	MMP4m	SHV-101
	C. freundii	CMP4b	TEM-2
	E. coli	CMP4c*	CTXM-9, OXA-10, *mcr-9*
	E. coli	CMP4d	CTXM-15, CTXM-125, TEM-214
	E. coli	CMP4e	CTXM-15, CTXM-126, TEM-206
	E. coli	CMP4f	SHV-12
	E. coli	CMP4g	SHV-12
	E. coli	CMP4h	CTXM-15
	E. coli	CMP4i	CTXM-15, CTXM-17, TEM-214
	K. pneumoniae	CMP4k	CTX-M-15, CTX-M-125, SHV-52, TEM-206
	E. coli	EMP4b	TEM-2
	E. coli	EMP4c*	KPC-2
	E. coli	EMP4d*	TEM-214, OXA-48
	E. coli	EMP4e*	CTXM-126, NDM-5
	P. mirabilis	CoMP4b	TEM-2
	K. pneumoniae	LMP4a	SHV-36
	P. mirabilis	LMP4b	TEM-2

a ARG(s), antibiotic resistance gene(s).

b *, Bacterial strains subjected to whole-genome sequencing.

For the MP1 wastewater samples, three bacterial strains were positive for ESBLs. One E. coli strain was positive for both CTX-M-1 and TEM. Another E. coli strain harbored TEM and a strain of K. pneumoniae had an SHV.

For the MP2 wastewater samples, nine ESBL-producing bacterial strains were isolated. Three strains of E. coli had a CTX-M-1 gene, and two strains of E. coli had a CTX-M-9 gene. In addition, six strains of E. coli, one strain of C. youngae, and one strain of P. mirabilis had the TEM gene. Moreover, one of the isolated E. coli strains was resistant to Ertapenem, and it was positive following the β-CARBA test (Bio-Rad, Hercules, CA). The KPC-2 gene was detected in this bacterial strain, which explains its production of carbapenemase.

For the MP3 wastewater samples, 10 bacterial isolates were positive for ESBLs. Two E. coli strains harbored the CTX-M-1 gene, three K. pneumoniae strains had a SHV gene, and six E. coli strains and one P. mirabilis strain had a TEM gene.

For the MP4 wastewater results, 20 bacterial strains were positive for ESBLs, and three bacterial strains were positive for carbapenemases. Four strains of E. coli and one strain of K. pneumoniae were positive for the CTX-M-1 gene. Five strains of E. coli and one strain of K. pneumoniae were shown to have a CTX-M-9 gene. For the SHV gene, three strains of E. coli and three strain of K. pneumoniae had this gene. In addition, nine strains of E. coli, two strains of P. mirabilis, one strain C. freundii, and one strain of K. pneumoniae were shown to have a TEM gene. As for the carbapenemases, three isolates of E. coli showed resistance to ertapenem. All of these strains were positive following the β-CARBA test, with one of them (EMP4c) harboring the KPC-2 gene, another strain (EMP4d) harboring the OXA-48 gene, and the third strain (EMP4e) harboring the NDM-5 gene. One strain of E. coli (CMP4c) was shown to harbor the OXA-10, CTX-M-9, and *mcr-9* genes. The *mcr-9* analysis is ongoing since this is the first time that this gene has been found in an E. coli strain isolated from wastewater in France (data not shown).

## DISCUSSION

This descriptive study is among the first to describe the various Gram-negative bacterial isolates identified in different water sites around Marseille, France and to report the presence of ARGs, most importantly carbapenemases such as NDM, KPC, and OXA-48.

Interestingly, our detection of common ESBL genes such as CTX-M and TEM β-lactamases in E. coli and E. cloacae strains came from the drinking water canal and a river (Table 1). Similar results were found in rivers located in the province of Sichuan in China, where CTX-Ms were found mostly in *Enterobacteriaceae* species (15). Another study showed a high detection of TEM genes present in *Enterobacteriaceae* strains isolated from rivers in Podhale in southern Poland (16). The canal in Marseille (W5) is a watercourse that passes through 36 communes in the city, and the Huveaune river (W7) passes through several communes. This may explain the presence of ESBL-producing bacteria in these aquatic sites, since they have a long course and so are bound to be contaminated.

On the other hand, all the wastewater sample isolates contained the CTX-M and TEM genes. Similar studies have reported the presence of CTX-M-1 and CTX-M-9 genes in E. coli from wastewater in Japan and the Netherlands (17, 18). CTX-M and TEM are the most frequently found ESBLs in humans (19, 20). Moreover, TEM genes have been detected in poultry meat samples (21) and in wastewater released into rivers (22). SHV genes were detected in the *Enterobacteriaceae* strains isolated from wastewater in Germany (23) and in the wastewater from several hospitals in South Africa (24). SHV-101 was found in one strain of E. coli and one strain of K. pneumoniae. To date, no description of this gene has been given.

We detected the KPC-2, NDM-5, and OXA-48 genes in different E. coli strains in this study. KPC-2 was described and detected in KPC-producing K. pneumoniae clinical strains isolated in Chilean hospitals (25), and the NDM-5 gene was detected from wastewater in Japan (26). The OXA-48 gene was described to be highly present in E. coli strains in Mediterranean countries (27). This gene was detected clinically in France (28) and also in animals, such as cats and dogs (29). The presence of these carbapenemases in Marseille wastewater is alarming due to their circulation in various sites across the city.

From the MP4 wastewater samples, we detected an *mcr-9* gene in a strain of E. coli (CMP4c; data not shown). This gene was first detected in Salmonella enterica serotype Typhimurium, isolated from a patient in Washington State, and it was shown to have the ability to lower susceptibility to colistin (30). It was detected in bacterial strains isolated exclusively from humans (5). This gene was not previously described in wastewater and has not yet been clinically detected in Marseille.

Marseille is a big city with at least three large university hospitals and several smaller ones. In addition, the port of this city is a major shipping, transport, and immigration portal in France. In terms of the MP4 wastewater, a high number of bacterial isolates were identified, since the separated sewers are mainly located near all the hospitals of the city. This may explain the abundance of ARG and is simultaneously of concern because it implies a high level of circulating ARGs, specifically ESBL, in this community. Since it is known that ESBL is quasi-endemic, the detection of carbapenemase and *mcr* genes in the sewers is a warning sign to monitor their carriage in healthy, nonhospitalized individuals in the community. Overall, a high prevalence of ARGs was found in separated sewers and wastewater in the city of Marseille, France.

### Conclusions and future perspectives.

We are aware there are some limitations to our work, such as the need to increase our sample size and sampling frequency for a more complete monitoring. However, that may be done at a later stage with dedicated policies and resources. In addition, for example, the Huveaune river and the Marseille canal are both sites which extend over a large area and cut through various communes. This may increase the risk of possible contamination of these waters, leading to our results.

Nonetheless, our brief surveillance period allowed us to identify ARGs that were not previously described in water, as well as to establish a current epidemiologic inventory of ARGs in the water in Marseille. ESBL-producing and carbapenem-resistant Gram-negative bacteria are usually implicated in severe infections in humans.

We highlight a significant circulation of antibiotic-resistant bacteria in ecosystems which are in close contact with human activities, implying possible emerging community outbreak. We are now facing a public health challenge that is best dealt with by continuous, local surveillance to anticipate the spread of MDR bacteria, especially under the One Health umbrella. A lot remains to be explored, starting with adapting routine surveillance of water sites as a tool to track antimicrobial resistance, and creating and revising existing water treatment policies.

## MATERIALS AND METHODS

### Sample collection.

We collected 14 water samples from different water sampling sites (W1 to W7) in Marseille, France (Fig. 1), between October and November 2020. These water sites included seawater (W1-Vieux Port, W2-Plage des Catalans, W3-Plage de L’Huveaune, and W6 L’Estaque), freshwater (W4-Ruisseau des Aygalades and W7-L’Huveaune), and drinking water from one of the major sources in Marseille (W5-Canal de Marseille). Canal de Marseille water is treated with ferric chloride to make a decantation, and it does not contain any disinfectant. One liter of water was collected randomly from each of these sites. Samples were collected in sterile flasks and were transferred immediately to the laboratory to be placed at −80°C for later use.

In addition, four different wastewater samples collected from Marseille in March and April 2021 (Fig. 1) were added to the study. Two types of sampling were performed: one was a grab sample, in which the water is collected once, and the other is a composite sample, in which the water is collected over a period of time, 24 h in this case. Two grab samples (MP1 and MP3) were collected by the Bataillon des Marins Pompiers de Marseille (BMPM) from two different districts of Marseille: the National and the Belle-de-Mai. The composite sampling of wastewater was done by automatic ASP-Station 2000 RPS20B vacuum samplers (Endress Hauser, Huningue, France). This type of sampler allows the filling of a refrigerated flask of 20 L per 24 h of wastewater collected from 8 a.m. to 8 a.m. This process was automated from the Marseille municipal sewage system (Service d’Assainissement Marseille Métropole [SERAMM]). Samples were drawn from a combined network (composed of central Marseille wastewater and rainwater) for MP1 and MP2 and from a separated network (sewage only, from the entire city) for MP3 and MP4.

The geographical distances between the samples are presented in Table S1 in the supplemental material. All samples were transferred to the IHU Méditerranée Infection by BMPM, at 4°C, and were processed 1 h after sampling.

### Sample filtration.

We filtered 1 L of each of the water samples using a filtration ramp device and 0.2-µm-pore-size membranes (Nuclepore Track-Etch polycarbonate membrane; Whatman, Buckinghamshire, UK). Filtration membranes were used for bacterial isolation. Each membrane was incubated in tryptic soy broth (TSB) tubes at 37°C for 72 h for bacterial enrichment.

For the wastewater samples, prefiltration was conducted for the grab sample from the National district (MP1). This was performed using filtration papers with 10- to 20-µm porosity (FPLIS33; Labelians, Nemours, France). This was followed by filtration using 5-µm (TMTP04700; Merck Millipore) and 0.1-µm (VCWP04700; Merck Millipore) filtration membranes. The 0.1-µm filtration membranes were stored at 4°C in sterile water. Membranes were incubated in TSB tubes at 37°C for 72 h.

The three other wastewater samples were centrifuged at 700 × *g* for 10 min. The supernatants were then filtered using 5-µm nitrocellulose membranes (TMTP04700; Merck Millipore). The 5-µm filtration membranes were stored at 4°C in sterile water. The membranes were incubated in TSB tubes at 37°C for 72 h.

### DNA extraction.

From the 14 water samples and the 4 wastewater samples, we prepared genomic DNA by incubating a volume of samples from the TSB tubes at 56°C overnight using a EZ1 DNeasy blood tissue kit (Qiagen GmbH, Hilden, Germany). DNA extraction was performed following the manufacturer’s protocol. We then performed real-time PCR to detect the presence of specific ARGs. The primers used were those encoding genes for ESBLs (TEM, SHV, CTX-a, and CTX-b), genes for carbapenemases (OXA-23, OXA-58, OXA-24, OXA-48, NDM, KPC, and VIM), and colistin-resistant genes (*mcr*-1 to *mcr*-5 and *mcr-*8) (Table 3).

**TABLE 3:** Primers used in real-time PCR and standard PCR

PCR type	Primer	Sequence (5′–3′)^*a*^	Reference
Real-time PCR	CTX-M-a	F: CGGGCRATGGCGCARAC R: TGCRCCGGTSGTATTGCC P: CCARCGGGCGCAGYTGGTGAC	36
	CTX-M-B	F: ACCGAGCCSACGCTCAA R: CCGCTGCCGGTTTTATC P: CCCGCGYGATACCACCACGC	36
	TEM	F: TTCTGCTATGTGGTGCGGTA R: GTCCTCCGATCGTTGTCAGA P: AACTCGGTCGCCGCATACACTATTCTCAGA	36
	SHV	F: TCCCATGATGAGCACCTTTAAA R: TCCTGCTGGCGATAGTGGAT P: TGCCGGTGACGAACAGCTGGAG	36
	VIM	F: CACAGYGGCMCTTCTCGCGGAGA R: GCGTACGTYGCCACYCCAGCC P: 6-FAM-AGTCTCCACGCACTTTCATGACGACCGCGTCGGCG-TAMRA	37
	NDM	F: GCGCAACACAGCCTGACTTT R: CAGCCACCAAAAGCGATGTC P: 6-FAM-CAACCGCGCCCAACTTTGGC-TAMRA	38
	KPC	F: GATACCACGTTCCGTCTGGA R: GGTCGTGTTTCCCTTTAGCC P: 6-FAM-CGCGCGCCGTGACGGAAAGC-TAMRA	39
	OXA-23	F: TGCTCTAAGCCGCGCAAATA R: TGACCTTTTCTCGCCCTTCC P: FAM-GCCCTGATCGGATTGGAGAACCA-TAMRA	40
	OXA-24	F: CAAATGAGATTTTCAAATGGGATGG R: TCCGTCTTGCAAGCTCTTGAT P: FAM-GGTGAGGCAATGGCATTGTCAGCA-TAMRA	40
	OXA-48	F: TCTTAAACGGGCGAACCAAG R: GCGTCTGTCCATCCCACTTA P: 6-FAM-AGCTTGATCGCCCTCGATTTGG-TAMRA	37
	OXA-58	F: CGCAGAGGGGAGAATCGTCT R: TTGCCCATCTGCCTTTTCAA P: FAM-GGGGAATGGCTGTAGACCCGC-TAMRA	40
	*mcr-1*	F: GCAGCATACTTCTGTGTGGTAC R: ACAAAGCCGAGATTGTCCGCG P: FAM-GACCGCGACCGCCAATCTTACC-TAMRA	41
	*mcr-2*	F: CTGTGCCGTGTATGTTCAGC R: TTATCCATCACGCCTTTTGAG P: VIC-TGACCGCTTGGGTGTGGGTA-TAMRA	42
	*mcr-3*	F: TGAATCACTGGGAGCATTAGGGC R: TGCTGCAAACACGCCATATCAAC P: FAM-TGCACCGGATGATCAGACCCGT-TAMRA	42
	*mcr-4*	F: GCCAACCAATGCTCATACCCAAAA R: CCGCCCCATTCGTGAAAACATAC P: FAM-GCCACGGCGGTGTCTCTACCC-TAMRA	42
	*mcr-5*	F: TATCCCGCAAGCTACCGACGC R: ACGGGCAAGCACATGATCGGT P: FAM-TGCGACACCACCGATCTGGCCA-TAMRA	42
	*mcr-8*	F: TCCGGGATGCGTGACGTTGC R: TGCTGCGCGAATGAAGACGA P: FAM-TCATGGAGAATCGCTGGGGGAAAGC-TAMRA	43
Standard PCR	CTX-M-1	F: CCCATGGTTAAAAAATCACTGC R: CAGCGCTTTTGCCGTCTAAG	36
	CTX-M-9	F: GCGCATGGTGACAAAGAGAGTGCAA R: GTTACAGCCCTTCGGCGATGATTC	36
	TEM	F: ATGAGTATTCAACATTTCCGTG R: TTACCAATGCTTAATCAGTGAG	42
	SHV	F: ATTTGTCGCTTCTTTACTCGC R: TTTATGGCGTTACCTTTGACC	42
	NDM	F: CATTTGCGGGGTTTTTAATG R: CTGGGTCGAGGTCAGGATAG	38
	KPC	F: ATGTCACTGTATCGCCGTCT R: TTTTCAGAGCCTTACTGCCC	38
	OXA-48	F: TTGGTGGCATCGATTATCGG R: GAGCACTTCTTTTGTGATGGC	44

a R, reverse; F, forward; P, probe.

### Bacterial culture.

We inoculated 20 μL of each of the TSB tubes on different types of agar: Columbia agar + 5% sheep blood, MacConkey agar, MacConkey agar + ertapenem (0.5 µg/mL), MacConkey agar + cefotaxime (1 µg/mL), and LBJMR (Lucie Bardet Jean-Marc Rolain) agar plates containing 4 µg/mL colistin and 100 µg/mL vancomycin (31). The used concentrations were based on the European Committee on Antimicrobial Susceptibility Testing (EUCAST) guidelines. We then incubated the plates for 24 h at 37°C. The colonies were identified using matrix-assisted laser desorption ionization–time of flight mass spectrometry (Bruker Daltonik, Bremen, Germany), as described previously (32).

### Antimicrobial susceptibility test.

We used the disc diffusion method (in line with the EUCAST recommendations) to determine the antibiotic susceptibility of growing bacteria. The keyhole effect between a third- or fourth-generation cephalosporin and clavulanic acid implied the detection of ESBLs. We performed the Etest method (bioMérieux, Marcy-l’Etoile, France) to determine the MICs of Imipenem and Ertapenem. We used the unitary MIC microdilution method (Biocentric, Bandol, France) to determine the MIC of colistin, and the β-CARBA test to determine the presence of carbapenemase.

### Molecular and genomic analysis.

We performed qPCR and standard PCR to confirm our results on the bacterial strains to investigate ESBL genes (CTX-M, SHV, and TEM), carbapenemase genes (NDM, VIM, KPC, OXA-23, OXA-24, OXA-48, and OXA-58), and colistin-resistant genes (*mcr-1*, *mcr-2*, *mcr-3*, *mcr-4*, *mcr-5*, and *mcr-8*) (Table 3). We then performed gene sequencing of the positive samples using a BigDye terminator cycle sequencing kit (Applied Biosystems, Foster City, CA). The NCBI reference sequence database was used to detect antibiotic-resistant genes present in the isolates. Table S2 shows the reference sequence numbers of the detected ARGs.

The genomes of five carbapenem-resistant E. coli strains were sequenced using MiSeq technology (Illumina, Inc., San Diego, CA) applying a paired-end strategy. The Galaxy Europe platform (https://usegalaxy.eu/) was then used for analysis. The SPAdes algorithm was used to assemble the genomes (33), and Prokka was used for their annotation (34). ARGs were identified using the Resfinder database with Abricate (Galaxy version 1.0.1) (35). The sequenced genomes have been deposited under BioProject number PRJNA856496 in the NCBI database. All sequenced bacterial strains are available from the CSUR collection at the IHU laboratory with specific CSUR numbers (Q6629, Q6631, Q6632, Q6633, and Q6634) for each sample, and they are stored at −80°C.

### Data availability.

The sequenced genomes have been deposited under BioProject number PRJNA856496 in the NCBI Database.
