# Supplementary material for: Epidemiological Description and Detection of Antimicrobial Resistance in Various Aquatic Sites in Marseille, France
Source: Microbiol Spectr. 2023 Mar 28;11(2):e01426-22. doi: 10.1128/spectrum.01426-22 (PMC10101087; doi:10.1128/spectrum.01426-22)
Supplement: Supplemental file 1 — Tables S1 to S3. Download spectrum.01426-22-s0001.pdf, PDF file, 0.05 MB [file spectrum.01426-22-s0001.pdf]

Table S1: The distances (km) between the sampling sites in Marseille, France.

Sampling Location	W1	W2	W3	W4	W5	W6	W7	MP1 and MP2	MP3 and MP4
W1	-	1.6	3.83	8.91	6.54	8.72	3.68	2.08	3.45
W2	1.6	-	3.74	9.26	7.73	8.37	3.94	3.64	4.11
W3	3.83	3.74	-	12.77	10.2	12.34	1	5.07	1.95
W4	8.91	9.26	12.77	-	5.05	4.02	12.56	8.62	12.19
W5	6.54	7.73	10.2	5.05	-	8.01	9.65	5.17	8.97
W6	8.72	8.37	12.34	4.02	8.01	-	12.48	9.57	12.4
W7	3.68	3.94	1	12.56	9.65	12.48	-	4.5	1.02
MP1 and MP2	2.08	3.64	5.07	8.62	5.17	9.57	4.5	-	3.62
MP3 and MP4	3.45	4.11	1.95	12.19	8.97	12.4	1.02	3.62	-

(-) not applicable

Table S2: Reference sequence numbers of the detected antibiotic resistance genes.

Antibiotic Resistance Gene		Reference sequence
Extended-spectrum class A beta-lactamase	CTX-M-3	NG_048979.1
	CTX-M-9	NG_049043.1
	CTX-M-15	NG_048935.1
	CTX-M-17	NG_048955.1
	CTX-M-27	NG_048976.1
	CTX-M-125	NG_048918.1
	CTX-M-126	NG_048919.1
	SHV-12	NG_050590.1
	SHV-160	NG_050036.1
	SHV-187	NG_050053.1
Broad-spectrum class A beta-lactamase	SHV-36	NG_050075.1
	SHV-52	NG_050090.1
	SHV-89	NG_050121.1
	SHV-101	NG_049991.1
	TEM-2	NG_050234.1
	TEM-206	NG_050238.1
A beta-lactamase	TEM-98	NG_050307.1
	TEM-214	NG_050247.1
	TEM-217	NG_050250.1
Subclass B1 metallo-beta-lactamase	NDM-5	NG_049337.1
Oxacillin-hydrolyzing class D beta-lactamase	OXA-10	NG_049393.1
Carbapenem-hydrolyzing class D beta-lactamase	OXA-48	NG_049762.1
Carbapenem-hydrolyzing class A beta-lactamase	KPC-2	NG_049253.1

Table S3: List of the identified Gram-negative bacteria isolated from the wastewater samples (N=114).

Membrane	Isolates	Isolate code	Antibiotic Resistance Genes
MP1	*E. coli*	MMP1a	TEM-206
	E. coli	CoMP1a	-
	E. coli	CMP1a	CTX-M-15, TEM-214
	K. pneumoniae	MMP1c	SHV-187
	P. mirabilis	MMP1b	-
	P. mirabilis	CoMP1b	-
	P. mirabilis	CMP1b	-
	P. mirabilis	EMP1a	-
	P. mirabilis	LMP1a	-
	P. aeruginosa	CMP1c	-
	P. aeruginosa	EMP1a	-
MP2	*C. youngae*	MMP2a	TEM-214
	C. youngae	MMP2b	-
	E. coli	MMP2c	-
	E. coli	MMP2d	-
	E. coli	MMP2e	-
	E. coli	MMP2f	TEM-214
	E. coli	CMP2a	CTX-M-27
	E. coli	CMP2b	CTX-M-27, TEM-214
	E. coli	CMP2c	CTX-M-3, TEM-98, KPC-24
	E. coli	CMP2d	CTX-M-15, TEM-214
	E. coli	CMP2e	CTX-M-15, TEM-214
	E. coli	CMP2f	TEM-206
	K. pneumoniae	MMP2h	-
	P. mirabilis	MMP2g	-
	P. mirabilis	CoMP2a	-
	P. mirabilis	LMP2b	TEM-217
	P. penneri	LMP2a	-
	P. vulgaris	CoMP2b	-
	A. baumannii	CMP2g	-
	A. baumannii	EMP2a	-
	P. aeruginosa	EMP2b	-
MP3	*C. freundii*	MMP3a	-
	C. freundii	MMP3b	-
	E. coli	MMP3c	TEM-206
	E. coli	MMP3d	TEM-214
	E. coli	MMP3e	-
	E. coli	MMP3f	TEM-206
	E. coli	MMP3g	-

	E. coli	MMP3h	-
	K. pneumoniae	MMP3i	SHV-89
	P. mirabilis	MMP3j	TEM-206
	P. mirabilis	MMP3k	-
	E. coli	MMP3l	TEM-206
	K. aerogenes	MMP3m	-
	E. cloacae	EMP3a	-
	P. mirabilis	LMP3a	-
	P. vulgaris	LMP3b	-
	P. alcalifaciens	LMP3c	-
	P. alcalifaciens	LMP3d	-
	C. freundii	CMP3a	-
	E. coli	CMP3b	CTX-M-15, TEM-206
	E. coli	CMP3c	CTX-M-15, TEM-206
	K. pneumoniae	CMP3d	SHV-12
	K. pneumoniae	CMP3e	SHV-160
	P. mirabilis	CoMP3a	-
	P. mirabilis	CoMP3b	-
	P. mirabilis	CoMP3c	-
	A. veronii	EMP3b	-
	A. veronii	EMP3c	-
	A. veronii	EMP3d	-
	P. aeruginosa	EMP3e	-
	P. aeruginosa	EMP3f	-
	H. alvei	LMP3e	-
	M. morganii	LMP3f	-
	M. morganii	CMP3f	-
	P. aeruginosa	CMP3g	-
MP4	*C. freundii*	MMP4a	-
	C. freundii	MMP4b	-
	C. freundii	MMP4c	-
	C. freundii	MMP4d	-
	C. youngae	MMP4e	-
	E. asburiae	MMP4f	-
	E. cloacae	MMP4g	-
	E. coli	MMP4h	-
	E. coli	MMP4i	TEM-206
	E. coli	MMP4j	TEM-206
	E. coli	MMP4k	TEM-206, NDM-5
	E. coli	MMP4l	SHV-101, TEM-214
	K. pneumoniae	MMP4m	SHV-101, KPC-24
	P. penneri	MMP4n	-

	C. freundii	CMP4a	-
	C. freundii	CMP4b	TEM-2
	E. coli	CMP4c	CTXM-9, OXA-10, *mcr-9*
	E. coli	CMP4d	CTXM-15, CTXM-125, TEM-214
	E. coli	CMP4e	CTXM-15, CTXM-126, TEM-206
	E. coli	CMP4f	SHV-12
	E. coli	CMP4g	SHV-12
	E. coli	CMP4h	CTX-M-15
	E. coli	CMP4i	CTXM-15, CTXM-17, TEM-214
	E. coli	CMP4j	-
	K. pneumoniae	CMP4k	CTX-M-15, CTX-M-125, SHV-52, TEM-206
	E. cloacae	EMP4a	-
	E. coli	EMP4b	TEM-2
	E. coli	EMP4c	KPC-2
	E. coli	EMP4d	TEM-214, OXA-48
	E. coli	EMP4e	CTXM-126, NDM-5
	E. coli	EMP4g	KPC-24
	P. mirabilis	CoMP4a	-
	P. mirabilis	CoMP4b	TEM-2
	K. pneumoniae	LMP4a	SHV-36
	P. mirabilis	LMP4b	TEM-2
	P. mirabilis	LMP4c	-
	P. mirabilis	LMP4d	-
	P. vulgaris	LMP4e	-
	P. alcalifacie	LMP4f	-
	P. rettgeri	LMP4g	-
	P. alcalifacie	LMP4j	-
	M. morganii	LM4h	-
	P. rettgeri	LM4i	-
	S. putrefaciens	EMP4f	-
	A. baumannii	EMP4h	-
	P. aeruginosa	EMP4i	-
	P. aeruginosa	EMP4j	-
	A. veronii	EMP4k	-

(-) No ARGs detected.
